# Improvement of Intradialytic Hypotension in Diabetic Hemodialysis Patients Using Vitamin E-Bonded Polysulfone Membrane Dialyzers

**DOI:** 10.1111/j.1525-1594.2012.01483.x

**Published:** 2012-10

**Authors:** Masahide Koremoto, Noriko Takahara, Masahiro Takahashi, Yasuhiro Okada, Kouhei Satoh, Tomoyoshi Kimura, Tomoyuki Hirai, Itaru Ebihara, Daisuke Nagasaku, Shigeo Miyata, Shunji Maniwa, Takuji Kouzuma, Tetsurou Arimura, Junzou Kamei

**Affiliations:** * Asahi Kasei Kuraray MedicalTokyo; † Hoshi UniversityTokyo; ‡ Asahi Kasei PharmaTokyo; § Ako City HospitalHyogo; ** Asahikawa-Kousei General HospitalHokkaido; †† Sendai Social Insurance HospitalMiyagi; ‡‡ Kesennuma City HospitalMiyagi; §§ Kouwa ClinicAomori; *** Mizushima Cooperative HospitalOkayama; ††† Mito Saiseikai General HospitalIbaraki; ‡‡‡ Yujin-Yamasaki HospitalShiga, Japan; §§§ Social Insurance Shiga HospitalShiga, Japan

**Keywords:** Blood pressure, Intradialytic hypotension, Plasma nitric oxide, Plasma peroxynitrite, Serum albumin, Vitamin E-bonded super high-flux polysulfone membrane dialyzer

## Abstract

Currently, there are no detailed reports on the effects of vitamin E-bonded polysulfone (PS) membrane dialyzers on intradialytic hypotension (IDH) in diabetic hemodialysis (HD) patients. This study was designed to evaluate changes in intradialytic systolic blood pressure (SBP) using “VPS-HA” vitamin E-bonded super high-flux PS membrane dialyzers. The subjects were 62 diabetic HD patients whose intradialytic SBP fell by more than 20%. Group A comprised patients who required vasopressors to be able to continue treatment or who had to discontinue therapy due to their lowest intradialytic SBP being observed at 210 min (28 patients). Group B comprised patients who showed no symptoms and required no vasopressors but showed a gradual reduction in blood pressure, with the lowest intradialytic SBP seen at the end of dialysis (34 patients). The primary outcome was defined as the lowest intradialytic SBP after 3 months using VPS-HA. Secondary outcomes included changes in the following: lowest intradialytic diastolic blood pressure, pulse pressure, pulse rate, plasma nitric oxide and peroxynitrite, serum albumin, and hemoglobin A1c. Group A's lowest intradialytic SBP had significantly improved at 3 months (128.0 ± 25.1 mm Hg vs. 117.1 ± 29.2 mm Hg; *P* = 0.017). Group B's lowest intradialytic SBP had significantly improved at 1 month (134.4 ± 13.2 mm Hg vs. 121.5 ± 25.8 mm Hg; *P* = 0.047) and 3 months (139.1 ± 20.9 mm Hg vs. 121.5 ± 25.8 mm Hg; *P* = 0.011). We conclude that VPS-HA may improve IDH in diabetic HD patients.

Intradialytic hypotension (IDH) is a common complication of hemodialysis (HD). Shoji et al. has reported a correlation between IDH and mortality in HD patients (1). According to a report by the Japanese Society for Dialysis Therapy, the following types of HD patients show the lowering systolic blood pressure (SBP) under HD by more than 20% account for 43.7% overall, 40.9% of patients with chronic glomerulonephritis, and 48.1% of patients with diabetic nephropathy (DN) (2). Furthermore, in the same study, HD patients who exhibit decreased SBP under HD by more than 20% account for 43.7% overall and 46.6% of patients aged 75 to 90 years old (2). The number of renal failure patients with diabetes mellitus as a primary diagnosis is increasing every year in Japan, as are their proportion of HD patients: 14.9% in 1990, 26.0% in 2000, and 35.8% in 2010 (3). It thus appears likely that, in Japan at least, the number of aging patients or patients with DN with symptomatic IDH will continue to grow.

Several interventional studies on IDH include comparison of albumin concentrations, comparison of the dialysate sodium concentration (4), comparison of the dialysate calcium concentration (5), comparison of acetic acid concentration (6), and comparisons between HD and hemodiafiltration as treatment methods (7). However, few studies include any comparison of dialyzer flux or advance over conventional dialyzers. Matsumura et al. reported that VPS-H vitamin E-bonded high-flux polysulfone (PS) membrane dialyzer (Asahi Kasei Kuraray Medical, Tokyo, Japan) improved the IDH of eight HD patients at a single center (8). However, there are few multicenter studies on diabetic HD patients with symptomatic IDH that investigate the effects of the dialyzers used.

We hypothesized that VPS-HA vitamin E-bonded super high-flux PS membrane dialyzer (Asahi Kasei Kuraray Medical), which has a higher permeability than VPS-H, might improve IDH in DN HD patients. In carrying out our study on DN HD patients who have IDH with comorbid symptoms, we focused on the treatment approach.

## PATIENTS AND METHODS

### Study population

Eligible to enter the study were adult patients who had been undergoing thrice-weekly HD treatments at nine facilities with an arteriovenous fistula as blood access and anuria with no renal function for a minimum of 1 year. All participants had shown decreased SBP under HD by more than 20% when using conventional dialyzers in the 3 months preceding enrollment. Their primary diagnosis was DN. The study was approved by the Ethics Committee at each facility. Subjects who had cardiovascular disease with Class III and IV symptoms, as defined by the New York Heart Association criteria, were excluded. All patients submitted their written informed consent before the start of the study.

### Study design

In this study, we defined IDH as intradialytic SBP falling by more than 20%. We recognized two patterns of IDH. In one, blood pressure decreased during HD with visible symptoms. Vasopressors, increased dosage of vasopressors, or saline were administered, and intradialytic blood pressure was raised or HD treatment was interrupted (defined as Group A). The other pattern was that blood pressure gradually decreased during HD without any symptoms, and post-treatment blood pressure fell by more than 20% (defined as Group B). We thus decided to divide the patients into two groups according to the pattern by which the blood pressure fell.

To eliminate the influence on the data by confounding factors, all patients maintained the same HD conditions before starting to use VPS-HA as the interventional dialyzer. We regarded the type and dosage of antihypertensive medication, the type and dosage of erythropoiesis-stimulating agents (ESAs), dry weight, HD dosage (except for HD sessions with symptoms), sodium and calcium concentration of the dialysate, ultrafiltration volumes, temperature of the dialysate, environmental temperature, and intradialytic feeding as confounding factors that might affect intradialytic blood pressure.

The HD conditions were as follows. HD sessions were performed thrice-weekly for 4 to 5 h at a blood flow rate of approximately 200 mL/min, conforming to the Japanese HD standard conditions. Dialysate with sodium, calcium, and acetate concentrations of approximately 140, 3, and 10 mEq/L, respectively, was supplied by a centrally supplied dialysate system at a flow rate of approximately 500 mL/min. The ultrafiltration rate (UFR) was managed using the UFR controller in the dialysis equipment. Every patient's intradialytic UFR was kept the same both pre- and post-study. The water for the preparation of the dialysate was purified using a reverse osmotic filter, an ultrafiltrate filter, and an endotoxin retention filter.

### Measurements and analyses

The results reported in our preceding article (8) suggested that the effects of vitamin E-bonded PS membrane dialyzer could be successfully investigated over a research period of 3 months. The observation period for this study was 3 months after switching to VPS-HA, including measurements of some parameters at 0 (before switching to VPS-HA) and at 1 and 3 months.

Data taken in response to intradialytic BP variations were recorded at every facility. The sphygmomanometer used was not changed during this study. The SBP, diastolic blood pressure (DBP), and pulse rate (PR) were measured every 30 min during HD. The pulse pressure (PP) was calculated from SBP and DBP every 30 min during HD. Mean BP (MBP) was calculated from the summation value of DBP and one-third PP every 30 min during HD. Blood pressure values were recorded for 300 min. Each patient had their intradialytic BP measured for 2 weeks, and the average value was designated the patient's BP. Next, the BP of the patient of each group was calculated and analyzed from the average value.

All blood samples were taken from the HD session at the beginning of the week.

Plasma NOx was adjusted using the following process. Two milliliters of blood was collected using evacuated tubes, and the plasma was separated in a refrigerated centrifuge (or a refrigerated sample was centrifuged). Plasma aliquots of 0.5 mL were quickly prepared and immediately frozen to −30°C. Some samples were packed into boxes with dry ice and sent frozen to the Department of Pathophysiology and Therapeutics School of Pharmaceutical Sciences at Hoshi University. Plasma nitric oxide (NO_2_^−^) and plasma peroxynitrite (NO_3_^−^) were measured by mixing plasma samples with Greiss reagent. The color absorbance of the resulting compound was measured using an ENO-20 HPLC Visible Detector (Eicom, Tokyo, Japan) (9). The combined value of NOx was recorded after measuring both NO_2_^−^ and NO_3_^−^.

Plasma glycoalbumin (GA) was adjusted using the following process. Three milliliters of blood was collected using evacuated tubes, and the plasma was separated in a refrigerated centrifuge (or a refrigerated sample was centrifuged). Plasma aliquots of 0.8 mL were quickly prepared and then immediately frozen to −30°C. Some samples were packed into boxes with dry ice and sent frozen to the Diagnostics Department of the Research and Development Group at Asahi Kasei Pharma (Shizuoka, Japan). The concentration of plasma GA was measured using a Lucica GA-L kit (Asahi Kasei Pharma, Tokyo, Japan) (10). The data are expressed as percentage GA, determined from the ratio of the measured GA to the total albumin concentration.

Serum albumin, blood glucose level, urea nitrogen, hemoglobin, and hemoglobin A1c (HbA1c) were measured at each facility.

### Outcome measures

According to the result of a precedent article (8), the primary outcome measure was defined as the lowest intradialytic SBP after 3 months using VPS-HA: the BP at 210 min in Group A and the BP at the end of HD session in Group B. Secondary outcome measures included changes in the following: lowest intradialytic DBP, intradialytic PP, intradialytic MBP, intradialytic PR, plasma NOx, plasma GA, serum albumin, blood glucose, and HbA1c.

### Statistical analyses

In our sample size calculation, we assumed that fewer than 10 patients would be withdrawn and 20 patients would thus remain as analytical subjects. We also assumed that at least 10 patients’ lowest SBP would rise more than 10 mm Hg; consequently, the group's lowest SBP would rise significantly over 3 months, the length of the study term. We therefore decided that each group should consist of 30 patients.

Statistical analyses were performed using XLSTAT (ver. 2008, Addinsoft SARL, Paris, France). Baseline characteristics were compared using Student's *t*-test and the chi-squared test. Primary and secondary outcome measures were compared using Student's *t*-test, the Wilcoxon signed-ranks test, or repeated-measures analysis of variance followed by a post hoc test. A two-tailed *P* < 0.05 was regarded as statistically significant.

## RESULTS

### Patient distribution and baseline characteristics

May to August 2007 served as the entry period. Patients were randomly enrolled until there were more than 30 patients in one group. We aimed to enroll 30 patients; however, one group contained 30 patients, the other group contained 35. Sixty-five HD patients were therefore enrolled at nine facilities. One group started with 30 patients and the other group started with 35 patients.

The baseline characteristics of study patients are shown in Table 1. Patient characteristics in Table 1 showed no difference between Group A and Group B, except that Group B's average age was higher than that of Group A (*P* = 0.090) and Group B's serum albumin tended to be higher than that of Group A (*P* = 0.089). For intradialytic blood pressure, we identified a different pattern of reduction in blood pressure between Group A and Group B (Table 2): intradialytic SBP and PP from 60 to 240 min in Group A were significantly lower than those in Group B, and intradialytic DBP and MBP at 300 min in Group A were significantly higher than those in Group B. Preparations were made to treat the intradialytic symptoms of more Group A patients than Group B patients (data not shown).

**TABLE 1:** Baseline characteristics of study patients

	Group A (*n* = 30)	Group B (*n* = 35)	*P* value
Gender (male/female)	16/14	22/13	0.437
Age (years)	60.6 ± 10.0	65.0 ± 11.5	0.090
Dialysis vintage (months)	46.3 ± 35.3	60.8 ± 38.7	0.240
Weight (kg)	54.5 ± 10.2	56.2 ± 10.2	0.660
SBP (mm Hg)	156.5 ± 31.3	155.3 ± 26.2	0.762
DBP (mm Hg)	78.9 ± 16.2	75.8 ± 11.6	0.164
PP (mm Hg)	77.9 ± 19.8	79.2 ± 16.5	0.647
MBP (mm Hg)	104.8 ± 21.2	79.2 ± 16.5	0.504
PR (beats/min)	80.6 ± 14.2	80.6 ± 4.5	1.000
GA (%)	27.1 ± 8.0	25.0 ± 6.4	0.277
sAlb (g/dL)	3.4 ± 0.3	3.5 ± 0.4	0.089
BG (mg/dL)	160.0 ± 68.4	158.1 ± 43.3	0.939
UN (mg/dL)	63.8 ± 11.4	136.7 ± 11.7	0.233
Hb (g/dL)	10.6 ± 0.9	10.5 ± 0.9	0.629
HbA1c (%)	6.4 ± 1.0	6.5 ± 1.0	0.737
NOx (nmol/L)	63.6 ± 35.3	62.9 ± 74.4	0.960

Values are mean ± SD.

Statistical testing of the baseline characteristics between Group A and Group B were performed using Student's *t*-test and the chi-squared test.

SBP, systolic blood pressure; DBP, diastolic blood pressure; PP, pulse pressure; MBP, mean blood pressure; PR, pulse rate; GA, glycoalbumin; sAlb, serum albumin; BG, blood glucose; UN, urea nitrogen; Hb, hemoglobin; HbA1c, hemoglobin A1c; NOx, total of plasma nitric oxide (NO_2_^−^) and plasma peroxynitrite (NO_3_^−^).

**TABLE 2:** Baseline characteristics of study patients for each blood pressure

	Dialysis time (min)	Group A	Group B	*P* value
SBP (mm Hg)	0	156.5 ± 31.3	155.3 ± 26.2	0.762
	30	142.2 ± 26.9	151.9 ± 33.5	0.138
	60	131.5 ± 27.5	149.0 ± 25.8	0.000*
	90	133.7 ± 27.1	148.5 ± 24.9	0.008*
	120	125.2 ± 24.6	149.3 ± 22.6	0.000*
	150	123.2 ± 24.2	142.3 ± 20.0	0.000*
	180	120.3 ± 29.5	145.0 ± 22.8	0.000*
	210	117.1 ± 29.2	143.2 ± 27.6	0.000*
	240	119.6 ± 31.9	145.6 ± 32.2	0.000*
	270	120.4 ± 41.4	128.2 ± 53.7	0.572
	300	144.2 ± 36.7	121.5 ± 51.1	0.085
DBP (mm Hg)	0	78.9 ± 16.2	75.8 ± 11.6	0.164
	30	74.2 ± 15.4	74.7 ± 10.5	0.872
	60	70.1 ± 14.6	74.4 ± 14.1	0.113
	90	71.6 ± 15.7	69.9 ± 11.5	0.573
	120	67.4 ± 16.2	75.3 ± 12.5	0.003*
	150	66.1 ± 13.7	72.5 ± 11.3	0.020*
	180	66.1 ± 17.1	73.7 ± 12.1	0.003*
	210	62.3 ± 12.9	71.6 ± 9.9	0.000*
	240	65.0 ± 21.3	73.7 ± 18.4	0.020*
	270	60.2 ± 19.4	66.3 ± 16.3	0.389
	300	82.2 ± 16.5	66.0 ± 19.8	0.033*
PP (mm Hg)	0	77.9 ± 19.8	79.2 ± 16.5	0.647
	30	67.8 ± 17.6	75.2 ± 18.7	0.105
	60	61.2 ± 17.5	73.9 ± 17.6	0.000*
	90	61.7 ± 17.5	77.0 ± 21.1	0.001*
	120	57.6 ± 13.9	73.3 ± 16.1	0.000*
	150	56.2 ± 14.9	69.2 ± 14.5	0.000*
	180	55.2 ± 18.2	70.9 ± 18.4	0.000*
	210	55.2 ± 22.2	71.7 ± 21.8	0.001*
	240	60.2 ± 20.5	72.6 ± 24.0	0.003*
	270	63.8 ± 32.8	64.8 ± 22.0	0.915
	300	62.0 ± 20.1	60.1 ± 20.2	0.790
MBP (mm Hg)	0	104.8 ± 21.2	79.2 ± 16.5	0.504
	30	96.9 ± 19.2	75.2 ± 18.7	0.875
	60	90.6 ± 18.9	73.9 ± 17.6	0.045*
	90	92.3 ± 19.5	77.0 ± 21.1	0.990
	120	86.7 ± 19.0	73.3 ± 16.1	0.001*
	150	85.2 ± 17.2	69.2 ± 14.5	0.239
	180	84.2 ± 21.2	70.9 ± 18.4	0.001*
	210	80.6 ± 18.4	71.7 ± 21.8	0.000*
	240	83.2 ± 24.8	72.6 ± 24.0	0.000*
	270	80.3 ± 26.7	64.8 ± 22.0	0.920
	300	102.8 ± 23.2	60.1 ± 20.2	0.023*
PR (beats/min)	0	80.6 ± 14.2	80.6 ± 4.5	1.000
	30	79.6 ± 16.7	78.8 ± 2.0	0.927
	60	78.0 ± 11.1	75.8 ± 4.0	0.718
	90	79.8 ± 18.5	77.6 ± 3.7	0.821
	120	79.6 ± 12.0	78.8 ± 3.4	0.901
	150	80.2 ± 10.2	80.2 ± 4.7	1.000
	180	92.8 ± 15.5	82.0 ± 4.3	0.218
	210	94.8 ± 14.4	81.2 ± 5.9	0.119
	240	92.2 ± 13.7	85.2 ± 8.9	0.417
	270	91.7 ± 16.8	88.0 ± 4.3	0.780

* *P* < 0.05

Values are mean ± SD.

Statistical testing for baseline characteristics between Group A and Group B was performed using Student's *t*-test.

SBP, systolic blood pressure; DBP, diastolic blood pressure; PP, pulse pressure; MBP, mean blood pressure; PR, pulse rate.

**FIG. 1:**
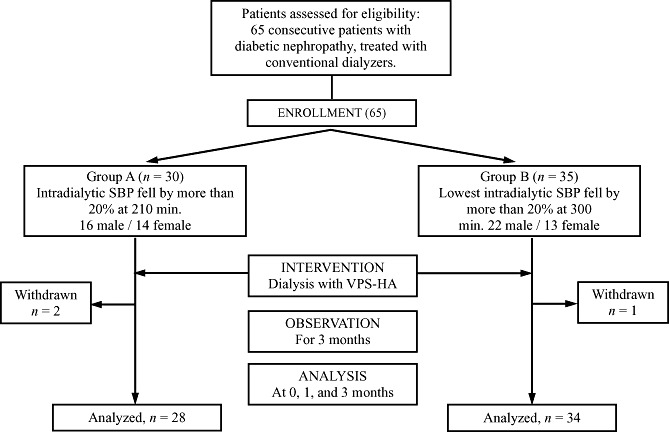
Study flow diagram. BP, blood pressure; SBP, systolic blood pressure.

After starting the study, three patients left, having withdrawn their agreement to take part. We thus adopted 62 patients as subjects for the final analysis: 28 in Group A and 34 in Group B. All these 62 patients continued with the study (Fig. 1).

### Pharmaceutical agents as confounding factors

Prior to the study, all the subjects were being administered one or more of the following antihypertensive medications: calcium channel blockers, angiotensin-converting enzyme inhibitors, alpha-blockers, beta-blockers, angiotensin receptor blockers, and other types. All patients maintained the same dosages and types of antihypertensive medications that they were being prescribed before the study began. As ESAs, either epoetin alpha or epoetin beta was used, and the same dosages and types of ESAs (alpha or beta) were used as before the start of the study.

### Conventional dialyzers

All the subjects had been undergoing HD using the following conventional dialyzers: APS-SA (PS, Asahi Kasei Kuraray Medical), APS-S (PS, Asahi Kasei Kuraray Medical), APS-MD (PS, Asahi Kasei Kuraray Medical), TS-U (PS, Toray, Tokyo, Japan), TS-UL (PS, Toray), CS-U (PS, Toray), PS-UW (PS, Kawasumi Laboratories, Inc., Tokyo, Japan), PES-S alpha (polyethersulfone, Nipro, Osaka, Japan), BP-H (polyethersulfone, JMS, Hiroshima, Japan), FDY-GW (polyethersulfone polymer alloy, Nikkiso, Tokyo, Japan), BG-PQ (polymethyl methacrylate, Toray), EK (ethylene vinyl alcohol, Kawasumi Laboratories, Inc.), kf-m (ethylene vinyl alcohol, Kawasumi Laboratories, Inc.), FB-U beta (cellulose triacetate, Nipro), FB-F (cellulose triacetate, Nipro), FB-E (cellulose triacetate, Nipro), AM-BC-P (cellulose, Asahi Kasei Kuraray Medical). Conventional PS membrane dialyzers were being used by 75% of the subjects in Group A and 65% in Group B.

### Blood pressure analysis

All the planned data were obtained from the analysis subjects, except for the PR of patients in Group A at 300 min, which was not recorded.

As for the intradialytic blood pressure of Group A patients (Table 3), some blood pressures at 3 months had risen significantly: SBP at 210 min: 117.1 ± 29.2 mm Hg at 0 months, 128.0 ± 25.1 mm Hg at 3 months (*P* = 0.017); DBP at 210 min: 62.3.1 ± 12.9 mm Hg at 0 months, 68.4 ± 15.0 mm Hg at 3 months (*P* = 0.012); MBP at 210 min: 77.8 ± 21.0 mm Hg at 0 months and 85.6 ± 21.2 mm Hg at 3 months (*P* = 0.014). Additionally, PR at 90 min at 1 month briefly but significantly rose (79.8 ± 18.5 beats/min at 0 months, 97.0 ± 12.8 beats/min at 1 month, *P* = 0.039).

**TABLE 3:** Variation in intradialytic blood pressure of Group A at pre- and post-intervention using VPS-HA

		0	1		3	
		
	Dialysis time (min)			*P* value		*P* value
SBP (mm Hg)	0	156.5 ± 31.3	159.5 ± 24.6	0.299	157.5 ± 27.1	0.264
	30	142.2 ± 26.9	134.0 ± 29.2	0.287	142.0 ± 28.5	0.589
	60	131.5 ± 27.5	132.0 ± 26.2	0.711	135.3 ± 30.1	0.376
	90	133.7 ± 27.1	134.3 ± 28.0	0.941	136.0 ± 32.2	0.530
	120	125.2 ± 24.6	126.7 ± 24.6	0.627	126.9 ± 26.6	0.898
	150	123.2 ± 24.2	122.1 ± 24.9	0.679	126.6 ± 27.2	0.408
	180	120.3 ± 29.5	119.8 ± 26.1	0.636	126.9 ± 28.3	0.142
	210	117.1 ± 29.2	115.9 ± 28.9	0.566	128.0 ± 25.1	0.017*
	240	119.6 ± 31.9	115.4 ± 29.1	0.278	135.6 ± 28.6	0.023*
	270	120.4 ± 41.4	121.9 ± 27.9	0.846	134.0 ± 33.4	0.100
	300	144.2 ± 36.7	132.1 ± 38.0	0.551	136.5 ± 33.3	0.880
DBP (mm Hg)	0	78.9 ± 16.2	81.4 ± 14.9	0.146	81.5 ± 15.0	0.139
	30	74.2 ± 15.4	70.7 ± 14.5	0.677	75.6 ± 16.4	0.112
	60	70.1 ± 14.6	68.4 ± 14.1	0.985	72.8 ± 15.7	0.343
	90	71.6 ± 15.7	74.1 ± 14.4	0.168	70.5 ± 15.7	0.971
	120	67.4 ± 16.2	67.9 ± 15.1	0.550	67.0 ± 14.4	0.701
	150	66.1 ± 13.7	67.0 ± 13.6	0.808	65.4 ± 15.5	0.312
	180	66.1 ± 17.1	64.7 ± 13.9	0.596	68.3 ± 14.6	0.432
	210	62.3 ± 12.9	68.1 ± 20.8	0.262	68.4 ± 15.0	0.012*
	240	65.0 ± 21.3	63.3 ± 16.0	0.384	72.1 ± 17.4	0.136
	270	60.2 ± 19.4	61.3 ± 15.6	0.542	64.0 ± 15.6	0.154
	300	82.2 ± 16.5	74.0 ± 14.4	0.525	75.8 ± 19.5	0.537
PP (mm Hg)	0	77.9 ± 19.8	77.9 ± 18.6	0.996	78.5 ± 19.4	0.826
	30	67.8 ± 17.6	64.8 ± 23.1	0.644	66.3 ± 20.3	0.698
	60	61.2 ± 17.5	64.1 ± 20.0	0.378	62.3 ± 18.6	0.580
	90	61.7 ± 17.5	61.3 ± 20.7	0.782	65.0 ± 20.4	0.354
	120	57.6 ± 13.9	60.3 ± 17.5	0.178	59.3 ± 18.0	0.734
	150	56.2 ± 14.9	56.0 ± 17.8	0.943	57.7 ± 17.7	0.822
	180	55.2 ± 18.2	55.4 ± 20.8	0.685	59.6 ± 18.8	0.134
	210	55.2 ± 22.2	52.0 ± 21.7	0.474	58.8 ± 18.1	0.360
	240	60.2 ± 20.5	55.7 ± 19.5	0.251	62.6 ± 19.8	0.759
	270	63.8 ± 32.8	62.9 ± 25.2	0.715	66.6 ± 20.2	0.365
	300	62.0 ± 20.1	55.7 ± 21.9	0.748	58.6 ± 20.4	0.896
MBP (mm Hg)	0	101.1 ± 26.0	103.5 ± 23.8	0.205	103.7 ± 25.1	0.205
	30	89.9 ± 27.9	84.6 ± 27.0	0.225	91.3 ± 28.0	0.241
	60	85.5 ± 25.3	84.3 ± 24.4	0.840	89.0 ± 26.3	0.338
	90	87.5 ± 25.5	88.1 ± 26.5	0.822	87.6 ± 26.8	0.739
	120	82.3 ± 24.4	82.6 ± 24.1	0.818	82.8 ± 23.8	0.850
	150	80.9 ± 22.6	80.3 ± 23.7	0.769	80.7 ± 23.9	0.357
	180	80.5 ± 24.5	78.5 ± 23.0	0.296	82.7 ± 25.9	0.236
	210	77.8 ± 21.0	82.6 ± 23.8	0.815	85.6 ± 21.2	0.014*
	240	82.3 ± 24.4	77.4 ± 23.5	0.014*	90.3 ± 23.7	0.167
	270	87.8 ± 19.6	82.1 ± 16.7	0.513	85.9 ± 19.3	0.391
	300	104.8 ± 21.0	93.4 ± 21.8	0.529	95.4 ± 24.3	0.490
PR (beats/min)	0	80.6 ± 14.2	89.0 ± 11.9	0.438	87.0 ± 8.9	0.719
		30	79.6 ± 16.7	88.0 ± 11.6	0.249	82.4 ± 11.4	0.590
		60	78.0 ± 11.1	93.2 ± 14.2	0.192	83.4 ± 8.7	0.396
		90	79.8 ± 18.5	97.0 ± 12.8	0.039*	85.2 ± 12.2	0.303
		120	79.6 ± 12.0	97.4 ± 11.2	0.131	87.2 ± 13.0	0.361
		150	80.2 ± 10.2	97.4 ± 10.8	0.115	88.2 ± 11.9	0.310
		180	92.8 ± 15.5	100.4 ± 16.2	0.107	87.8 ± 9.3	0.213
		210	94.8 ± 14.4	97.5 ± 10.2	0.954	91.2 ± 10.7	0.505
		240	92.2 ± 13.7	88.0 ± 6.7	0.738	88.6 ± 11.6	0.499
		270	91.7 ± 16.8	107.5 ± 10.5	0.356	95.7 ± 12.8	0.751

* *P* < 0.05

Values are mean ± SD.

Statistical testing of either 0 month versus 1 month or 0 month versus 3 months was performed using repeated-measures ANOVA followed by a post hoc test.

SBP, systolic blood pressure; DBP, diastolic blood pressure; PP, pulse pressure; MBP, mean blood pressure; PR, pulse rate.

As for the intradialytic blood pressure of Group B patients (Table 4), some blood pressures at 3 months had risen significantly: SBP at 300 min: 121.5 ± 25.8 mm Hg at 0 months, 134.4 ± 13.2 mm Hg at 1 month (*P* = 0.047), and 139.1 ± 20.9 mm Hg at 3 months (*P* = 0.011); DBP at 300 min: 66.0 ± 19.8 mm Hg at 0 months, 67.7 ± 19.3 mm Hg at 1 month (*P* = 0.034), and 73.1 ± 19.3 mm Hg at 3 months (*P* = 0.016); MBP at 300 min: 84.5 ± 20.9 mm Hg at 0 months, 89.9 ± 16.2 mm Hg at 1 month (*P* = 0.022), and 95.1 ± 18.8 mm Hg at 3 months (*P* = 0.003).

**Table 4:** Variation in intradialytic blood pressure of Group B at pre- and post-intervention using VPS-HA

		0	1		3	
		
	Dialysis time (min)			*P* value		*P* value
SBP (mm Hg)	0	155.3 ± 20.1	157.7 ± 19.8	0.305	161.3 ± 24.3	0.031*
	30	151.9 ± 22.9	151.7 ± 22.9	0.978	157.0 ± 27.8	0.256
	60	149.0 ± 23.5	150.4 ± 22.7	0.612	156.6 ± 21.8	0.013*
	90	148.5 ± 23.0	153.2 ± 26.7	0.249	153.7 ± 23.8	0.266
	120	149.3 ± 22.5	148.5 ± 22.0	0.774	146.2 ± 22.0	0.460
	150	142.3 ± 20.3	139.8 ± 24.9	0.802	140.3 ± 24.7	0.667
	180	145.0 ± 22.6	144.3 ± 24.5	0.861	145.3 ± 26.6	0.888
	210	143.2 ± 25.5	135.0 ± 28.2	0.225	138.1 ± 26.8	0.306
	240	145.6 ± 30.2	142.0 ± 27.7	0.362	140.6 ± 26.7	0.148
	270	128.2 ± 31.3	133.9 ± 22.1	0.978	136.8 ± 18.8	0.261
	300	121.5 ± 25.8	134.4 ± 13.2	0.047*	139.1 ± 20.9	0.011*
DBP (mm Hg)	0	75.8 ± 11.6	76.6 ± 15.9	0.622	78.1 ± 17.0	0.186
	30	74.7 ± 10.5	72.9 ± 9.4	0.570	72.9 ± 13.8	0.882
	60	74.4 ± 14.1	75.6 ± 14.5	0.747	78.7 ± 12.4	0.026*
	90	69.9 ± 11.5	74.4 ± 13.1	0.071	73.4 ± 12.7	0.108
	120	75.3 ± 12.5	74.2 ± 11.8	0.563	72.9 ± 11.9	0.230
	150	72.5 ± 11.3	68.3 ± 13.3	0.591	69.2 ± 14.0	0.458
	180	73.7 ± 12.1	71.9 ± 11.3	0.189	73.8 ± 12.8	0.662
	210	71.6 ± 9.9	69.6 ± 12.9	0.226	72.5 ± 10.8	0.948
	240	73.7 ± 18.4	72.9 ± 13.4	0.643	73.0 ± 12.5	0.538
	270	66.3 ± 16.3	78.1 ± 13.0	0.054	76.8 ± 7.8	0.044*
	300	66.0 ± 19.8	67.7 ± 19.3	0.034*	73.1 ± 19.3	0.016*
PP (mm Hg)	0	79.2 ± 16.5	80.8 ± 16.9	0.430	82.2 ± 22.3	0.191
	30	75.2 ± 18.7	78.3 ± 17.6	0.523	82.1 ± 23.6	0.199
	60	73.9 ± 17.6	74.6 ± 17.3	0.484	77.3 ± 17.1	0.056
	90	77.0 ± 21.1	76.7 ± 18.3	0.994	78.0 ± 17.4	0.814
	120	73.3 ± 16.1	75.2 ± 18.3	0.301	72.4 ± 17.9	0.942
	150	69.2 ± 14.5	70.4 ± 17.9	0.402	69.7 ± 16.6	0.914
	180	70.9 ± 18.4	73.1 ± 18.9	0.410	70.7 ± 19.9	0.885
	210	71.7 ± 21.8	67.3 ± 23.8	0.884	65.4 ± 20.8	0.257
	240	72.6 ± 24.0	69.7 ± 21.2	0.355	67.2 ± 20.1	0.081
	270	64.8 ± 22.0	53.1 ± 11.0	0.105	60.6 ± 16.3	0.375
	300	60.1 ± 20.2	60.6 ± 14.0	0.942	63.7 ± 16.1	0.612
MBP (mm Hg)	0	98.6 ± 20.7	100.0 ± 22.5	0.420	101.9 ± 24.4	0.068
	30	88.7 ± 31.3	88.9 ± 28.5	0.991	90.2 ± 31.0	0.468
	60	94.6 ± 24.2	95.8 ± 23.9	0.554	99.6 ± 24.1	0.008*
	90	87.6 ± 26.8	92.3 ± 28.9	0.079	91.9 ± 28.1	0.114
	120	95.9 ± 22.7	95.5 ± 21.2	0.971	93.2 ± 21.6	0.293
	150	87.2 ± 27.0	84.4 ± 26.5	0.875	85.9 ± 26.6	0.499
	180	93.4 ± 21.8	92.4 ± 21.8	0.415	93.4 ± 23.7	0.687
	210	93.4 ± 17.7	85.1 ± 25.7	0.223	89.4 ± 23.6	0.558
	240	97.5 ± 19.6	92.9 ± 21.9	0.512	93.4 ± 20.4	0.282
	270	87.0 ± 20.9	96.7 ± 15.7	0.131	96.8 ± 9.1	0.071
	300	84.5 ± 20.9	89.9 ± 16.2	0.022*	95.1 ± 18.8	0.003*
PR (beats/min)	0	80.6 ± 4.5	73.6 ± 21.0	0.500	81.6 ± 3.9	0.600
	30	78.8 ± 2.0	71.1 ± 22.0	0.400	76.4 ± 8.1	0.497
	60	75.8 ± 4.0	69.5 ± 20.0	0.693	77.6 ± 7.3	0.508
	90	77.6 ± 3.7	71.1 ± 21.0	0.665	79.0 ± 9.7	0.703
	120	78.8 ± 3.4	71.9 ± 21.0	0.614	80.6 ± 9.7	0.609
	150	80.2 ± 4.7	74.7 ± 22.0	0.404	83.6 ± 8.8	0.447
	180	82.0 ± 4.3	75.0 ± 22.0	0.569	83.4 ± 10.7	0.725
	210	81.2 ± 5.9	76.2 ± 22.0	0.213	84.0 ± 10.9	0.452
	240	85.2 ± 8.9	78.7 ± 23.0	0.825	84.6 ± 11.6	0.854
	270	88.0 ± 4.3	90.3 ± 8.0	0.606	89.0 ± 9.6	0.874
	300	87.7 ± 4.5	88.7 ± 7.0	0.580	91.7 ± 8.7	0.314

* *P* < 0.05

Values are mean ± SD.

Statistical testing of either 0 month versus 1 month or 0 month versus 3 months was performed using repeated-measures ANOVA followed by a post hoc test.

SBP, systolic blood pressure; DBP, diastolic blood pressure; PP, pulse pressure; MBP, mean blood pressure; PR, pulse rate.

As for symptomatic treatment of Group A patients, we monitored the number of times that treatment was required using saline infusion, glucose infusion, norepinephrine hydrochloride administration, or amezinium methylsulfate administration as the required vasopressor (data not shown). Symptomatic treatments per HD session tended to show a decreasing tendency (not significant). Saline: 0.08 ± 0.16 times at 0 months, 0.04 ± 0.08 times at 1 month, and 0.00 ± 0.00 times at 3 months; glucose: 1.58 ± 1.94 times at 0 months, 0.00 ± 0.00 times at 1 month, and 0.00 ± 0.00 times at 3 months; norepinephrine hydrochloride: 1.39 ± 1.15 times at 0 months, 1.00 ± 1.11 times at 1 month, and 0.89 ± 1.28 times at 3 months; amezinium methylsulfate: 1.20 ± 0.76 times at 0 months, 1.04 ± 0.70 times at 1 month, and 0.76 ± 0.69 times at 3 months. As for Group B patients’ symptomatic treatment, no difference was seen between pre- and post-intervention using VPS-HA.

### Blood sample analysis

For some secondary outcome measurements except for blood pressure, no significant differences between Group A and Group B (Table 5A) were seen. However, serum albumin in Group A had significantly risen from 3.4 ± 0.3 g/dL at 0 months to 3.6 ± 0.3 g/dL at 3 months (*P* = 0.006 in Table 5B); that of Group B had risen significantly from 3.5 ± 0.4 g/dL at 0 months to 3.6 ± 0.3 g/dL at 1 month (*P* = 0.042); and plasma NOx in Group A had risen significantly from 63.6 ± 35.3 nm at 0 months to 112.4 ± 85.4 nm at 3 months (*P* = 0.006).

**TABLE 5A:** Clinical characteristics of each group using VPS-HA (between groups)

	VPS-HA treatment period (months)	Group A	Group B	*P* value
Glycoalbumin (%)	0	27.1 ± 8.0	25.0 ± 6.4	0.277
	1	26.9 ± 6.7	25.1 ± 5.7	0.271
	3	26.8 ± 5.6	25.0 ± 7.0	0.291
Serum albumin (g/dL)	0	3.4 ± 0.3	3.5 ± 0.4	0.089
	1	3.5 ± 0.4	3.6 ± 0.3	0.059
	3	3.6 ± 0.3	3.6 ± 0.4	0.846
Blood glucose (mg/dL)	0	160.0 ± 68.4	158.1 ± 43.3	0.939
	1	152.4 ± 39.5	165.6 ± 52.5	0.456
	3	131.3 ± 37.3	152.0 ± 46.9	0.214
Urea nitrogen (mg/dL)	0	63.8 ± 11.4	136.7 ± 11.7	0.233
	1	64.7 ± 20.0	215.5 ± 14.7	0.204
	3	66.6 ± 21.9	175.1 ± 13.2	0.263
Hemoglobin (g/dL)	0	10.6 ± 0.9	10.5 ± 0.9	0.629
	1	10.4 ± 1.1	10.6 ± 0.9	0.743
	3	11.0 ± 1.1	10.6 ± 1.0	0.450
HbA1c (%)	0	6.4 ± 1.0	6.5 ± 1.0	0.737
	1	6.2 ± 1.3	6.4 ± 0.8	0.713
	3	6.2 ± 1.5	6.5 ± 0.9	0.543
Plasma NOx (nmol/L)	0	63.6 ± 35.3	62.9 ± 74.4	0.960
	1	78.7 ± 61.6	67.0 ± 60.1	0.463
	3	112.4 ± 85.4	85.5 ± 81.2	0.220

Values are mean ± SD.

Statistical testing between Group A and Group B was performed using Student's *t*-test.

**TABLE 5B:** Statistical differences in the P value of each group using VPS-HA (continuous variation)

	Group A		Group B	
		
	0 versus 1	0 versus 3	0 versus 1	0 versus 3
Glycoalbumin (%)	0.766	0.794	0.951	0.993
Serum albumin (g/dL)	0.146	0.006*	0.042*	0.333
Blood glucose (mg/dL)	0.751	0.280	0.502	0.635
Urea nitrogen (mg/dL)	0.879	0.609	0.196	0.749
Hemoglobin (g/dL)	0.657	0.379	0.436	0.466
HbA1c (%)	0.564	0.579	0.303	0.788
Plasma NOx (nmol/L)	0.232	0.006*	0.698	0.097

* *P* < 0.05

“0 versus 1” means the *P* value between each data item at 0 months and each data item at 1 month.

“0 versus 3” means the *P* value between each data item at 0 months and each data item at 3 months.

Values are mean ± SD.

Statistical testing of each group was performed using repeated-measures ANOVA followed by a post hoc test.

## DISCUSSION

The results of our present study, lasting 3 months, which included 62 Japanese HD patients with DN as their primary diagnosis, indicated that using VPS-HA vitamin E-bonded super high-flux PS membrane dialyzer significantly increased the lowest intradialytic SBP and DBP, and also improved IDH. Specifically, the lowest intradialytic SBP in Group A rose without the need for additional dosages of vasopressor or symptomatic treatment. Although the rate of decrease of SBP was significant, the rate of decrease improved after the start of the study. The rate of decrease of DBP also improved. When Group A's patients showed the lowest BP and the condition of the patients at 210 min was serious before using VPS-HA, vasopressor was administered and BP rose as a result at the end of dialysis. A phenomenon in Group A was also observed in which BP rose rapidly at 300 min before using VPS-HA. Similarly, in Group B, when there was no rapid fall in BP, there was no need to use vasopressors, etc., and the rise of the rapid blood pressure at the end of the session was not seen. The significant improvement in BP after switching to the vitamin E-bonded PS membrane dialyzers resulted in a reduction in the use of vasopressors or similar medications during dialysis. Our present study showed that VPS-HA has the potential to improve IDH in diabetes dialysis patients.

In our present study, dialyzer flux, which influences the removal performance of free immunoglobulin light chains (11) was almost the same before and after intervention. As for the absorbance potential of protein-bound solutes by the inner surfaces of the membrane, conventional polymethyl methacrylate dialyzers were used in only four patients and polyacrylonitrile in none; we therefore concluded that the adsorbance potential of the membrane would have little influence on our present study data (12,13). In addition, we concluded that the influence of IDH caused by bradykinins would be minimal, due to the use of conventional dialyzers.

Our present study showed that VPS-HA significantly improved the lowest intradialytic blood pressure and serum albumin concentration in the same patients. The membrane flux for each group during the prestudy, and that between pre- and post-intervention in both groups, was not significantly different. We thus concluded that the membrane flux of VPS-HA had no influence on the results of the present study.

Daugirdas reported that NO influences the vasodilatory effect and is one cause of IDH (14). Nitric oxide is believed to have two different physiological effects on the blood vessels. One is the relaxation and expansion of blood vessels when exposed to high levels of NO, resulting in a rapid fall in BP, and the other is contraction/expansion mediated by the release of NO from vascular endothelial cells. Because we judged that it was not practical to collect blood every 30 min, we decided to evaluate the contraction/expansion of the vascular endothelium. We therefore collected blood before the start of dialysis and measured NOx as reactants of NO. NOx levels were measured in our present study, and proved to be significantly elevated in Group A at 3 months and in Group B at 1 month. As for the observations of significant increases in the lowest intradialytic blood pressure and in NOx, it is possible that both vasodilation and vasoconstriction potency were improved, leading to alleviation of IDH. Miyazaki et al. reported their evaluation of endothelial function, estimated by flow-mediated vasodilation during reactive hyperemia using high-resolution ultrasound Doppler echocardiography, before and after a single session in patients on maintenance HD, using a vitamin E-bonded low-flux cellulose membrane dialyzer and a non-vitamin E-bonded PS membrane dialyzer. Their results indicated that HD using the low-flux cellulose type vitamin E-bonded membrane prevented HD-induced endothelial dysfunction and increases in oxidized LDL (15). In our present study, a transient increase in PR was observed in Group A at 1 month, possibly due to the influence of improved contractile/diastolic capacity of the blood vessels.

We did not measure increased amount of food ingested or any inflammation markers. In light of the increasing improvement of albumin concentration as a result of using vitamin E-bonded membrane dialyzers, further research on serum albumin as a factor in colloid osmotic pressure would be worthwhile.

Davenport et al. reported that DN HD patients tended to be prescribed higher dosages of antihypertensive medications, and showed worse compliance with blood pressure guidelines, higher weight gain in interdialysis, and more episodes of IDH than non-DN HD patients (16). The serious nature of these problems prompted us to investigate the effect on IDH in DN HD patients by testing a single variable, namely the use of vitamin E-bonded super high-flux PS membrane dialyzers.

We performed a subanalysis of dialyzers pre- and post-study, which showed that APS-SA was the most commonly used. Thirteen patients in Group A used APS-SA. In our comparative review of pre- and post-study results in this patient group, no significant difference due to the use of different dialyzers was observed.

The number of DN HD patients has been markedly increasing in Japan, and the importance of medical management of such patients has grown accordingly. Our group reported, in a previous study, improved IDH symptoms in eight HD patients in a single facility (8). The results of the present study, showing improved IDH in DN HD patients and a decreased management burden on medical staff, will be useful in HD therapy.

## CONCLUSION

The VPS-HA vitamin E-bonded super high-flux PS membrane dialyzer appears to improve the intradialytic hypertension of diabetic nephropathy patients receiving hemodialysis. Randomized controlled trials on other potential effects of vitamin E-bonded membrane dialyzers will now be needed.
